# Supplementary material for: Patterns of Intron Gain and Loss in Fungi
Source: PLoS Biol. 2004 Nov 30;2(12):e422. doi: 10.1371/journal.pbio.0020422 (PMC532390; doi:10.1371/journal.pbio.0020422)
Supplement: Table S1 — Also available at http://genes.mit.edu/NielsenEtAl/. (4.3 MB ZIP). [file pbio.0020422.st001.zip › NielsenEtAl/html/611.html]

AN7723.1.NCU01370.1.MG09487.1.FG06122.1


```
 CLUSTAL W (1.82) Multiple Sequence Alignments - Introns Inserted


Sequence 1: NCU01370.1	603 aa
Sequence 2: FG06122.1	577 aa
Sequence 3: MG09487.1	616 aa
Sequence 4: AN7723.1	553 aa
Alignment Length: 638 aa
Number Identitical Residues: 160 aa
Alignment Score (without introns) 8651


MG09487.1 	MRTRNSLGELPTSPSEEQTSAKKRGHAEDEPQVEKDLTPRKRRKPTRFRESPQDTRKKPA
NCU01370.1	MPPRRKSTDQHDLVERPSR-KRPLPADPYEVPDEDDAVPSAKRQ--RRQPEDGGTPSKRN
FG06122.1 	MP-RKKAVEESDSQQLPTRSLRKRNHQELEAIPESDQEETSPVK--RQRSVSHESPSTNG
AN7723.1  	MKRKHPEVDDEPSISTPKRQRISKTNGHVNGEGSPEPPPSKRVK---------VTPQKPA
          	*  :.   :     .    .         :   . :       :          : ..  

MG09487.1 	TAPKAVVEPKAKEEDGRPPKRNASDLIGDAQEEPAQTPTKRKPGRPKGSKNLPKPPLNGI
NCU01370.1	ATPRKQVDFLQRDLASVPE---EPESPTEQTP------SKKRIGRP--PKAKTGGTPSGK
FG06122.1 	ETNGNINGFMSNGAEDVAAS--ESESLSKVDPEETPKPAPKRRGRP--PKKAVN---AGS
AN7723.1  	AETAATLKA------------------------------------------------SGL
          	                                                          * 

MG09487.1 	HTTPSKSQSGRKWATPIKTNGPNGIDTPRRRNLADRSARKKSVSTMIERVVSGMMSDDEA
NCU01370.1	PSTPSALRKKQVDETPIKLKGISGVDTPGRRGIADRSARRKSARALIDRVLEGAVSDDED
FG06122.1 	P-TPKASR-TALFETPTKARFGLNPDTPGG---ADRSAKRKSTRALIEHVVGDDLTDEE-
AN7723.1  	RTPSNRSKAKALFSTSTSEK---GASTPTKVR-ADRSAKKKSAQLLLDQVEDSWEGGDQL
          	 :...  :      *. . .   . .**     *****::**.  ::::*  .   .:: 

MG09487.1 	GDDVLAREIYQSSEDEDDEDGGQQIPTVEVEAPAEDLETGEQSEVAPTPTRRRPGRPKGS
NCU01370.1	EEQQIAREIYESSEEEEEGDTQQEN-----------LADQEAELAATTPSKTGRGRGRGG
FG06122.1 	EYDGLAHKIYESSEDEDALEGDNAI-----------SAEASGLDEAATPSKSTPRRKAQR
AN7723.1  	AEEILAGEDAPDTIDRQRNDIVETV------------EADSTEPKQDKTEKTQPAPKRRA
          	  : :* :   .: :.:  :  :                 .      .. :         

MG09487.1 	GKNGPAR-KRSPTPPRDLPPYERYFFQNKPGSLKTSGNRLN-MELLTHEEYFNLVRDYK-
NCU01370.1	RPPGLARRKKSPTPPRDLPPHEQYFYQNKPGSNKTSNNTLSSLDLLIHEEYFSLLRNLGQ
FG06122.1 	KKP-----ARSPTPPRDLPPHELYFAHNKPGRAKTSNNTLGSLALLTHDEYFTIVRETS-
AN7723.1  	GRPKGAKNKRSPTPEGDLPPHERYFFQNRAGPPRTSNNNLNKVNLLTHEEYFDKHANYQ-
          	     :   :****  ****:* ** :*:.*  :**.* *..: ** *:***    :   

MG09487.1 	DPHVSDVTFLQNMHADSFRQWAFELSQGFSICLYGYGSKRALLQRFATELYHRG------
NCU01370.1	DPHEKHIDALQSQHAASFPQWAFELSQGFSVCLYGYGSKRRLLHQFAEYLFSSSGGNDTN
FG06122.1 	DHHEGDIEFLESLHAESFPQWAFELSQGFNLCLYGYGSKRRLLHKFAGHLHSRS--RKEK
AN7723.1  	DPCKRETAFLNDVHRRSFPQWNFEFSQGFNICLYGYGSKRRLTQGFADWLYRRHS---SA
          	*    .   *:. *  ** ** **:****.:********* * : **  *.   .     

MG09487.1 	HKRIVVVNGYVRTSGGIKEALSTMARAVDPSFRLPAT--LPVTMAQGLTALLADHP-ETV
NCU01370.1	NTTIIMINGHTRTLT-FREILTTISAAIDPTFRLPSG--NPLAMIQNLFTLLSSFAQKPR
FG06122.1 	GDKIVIINGYAPNTT-MREILSTIGSAVDPAHRIPLT--QPAVMVPAILSHLNTTS--ST
AN7723.1  	PPSVVIVNGHTPNLS-IRSIFATIVTAVLGADIPSKMGAQPVEVLELLQSALRSRPDQDP
          	   ::::**:. .   ::. ::*:  *:  :   .  .: *  :   : : *   ...  

MG09487.1 	LTIIVNSIDAP--GMRKGGSSNTTQAILAQLASHKQIRLVCSADTPDFSLLWDAGQRSQF
NCU01370.1	ITLLLASLDSPFSPLRKPQ----TQSLLAHLSSHPAISLACTVDTPDFPLLWDSSLRSSF
FG06122.1 	ITLIVNSIDA--APLRKPG----SQTALAQLAAHPHIRLVCSADTPDFALLWDIGVRSAF
AN7723.1  	ITVFINSIDAP--SLRRAT----NQALLARLAATPKIHLLVTADTPNFLLMWDISLRDQF
          	:*::: *:*:.   :*:      .*: **:*::   * *  :.***:* *:** . *. *

MG09487.1 	NFAMHDCTTFAPFGAAELDVVDEVHELLGRKDRRVGGKDGVIFVLRSLPENAKNLFRLLV
NCU01370.1	NFAFHDCTTFADH-SKELDVVDEVHELLGRMGKRAGGKEGVAFVLRSLPENARKLFGLIV
FG06122.1 	NMGFHDCTTFAPY-TAELDVVDEVHELLGRNAHRVNGREGVAFVLRSLPENAKNLFRLLV
AN7723.1  	NFVFHDCTTFTPF-DAEFDVVEEVHSLLGRKGRRVGGKEGVEFVLKSLPENAQNLYRVLL
          	*: :******: .   *:***:***.****  :*..*::** ***:******::*: :::

MG09487.1 	TEALVAMEEGSGMGMG------DFLGA-DGPGVEYRMMYNKAVEEFICSSEMAFRTLLKE
NCU01370.1	GEVLVAFEDGGGGGGGTSGAGDFAAGG-EGPGVEYRMLYNKAVEEFICSSEMAFRTLLKE
FG06122.1 	GEVLIAIEEEGDGGD-------------EPTGIEYKMVYNKAVEEFICSSEMAFRTLLKE
AN7723.1  	TELLSMMDEYLNGEDDMDNGDGAADGPKDEPGIEFRALYQKASEEFIASSEMMFRTLLKE
          	 * *  :::  .   . .....   . .: .*:*:: :*:** ****.**** *******

MG09487.1 	2FHDHQIITSRKDTLGTELLCLPFRKEEIEGILEDLMG-
NCU01370.1	2FHDHQIITSHKDSIGTEYLSLPFRKEELESILEELMS-
FG06122.1 	2FHDHQIITSMKDALGTELLSLPFRKDELEAILEDLTS-
AN7723.1  	~FHDHQMITSRMDASGMEILSVPLSRNEMEGVLEDLVLG
          	 *****:***  *: * * *.:*: ::*:*.:**:*  .
```
